# A model-based assay design to reproduce in vivo patterns of acute drug-induced toxicity

**DOI:** 10.1007/s00204-017-2041-7

**Published:** 2017-08-29

**Authors:** Lars Kuepfer, Olivia Clayton, Christoph Thiel, Henrik Cordes, Ramona Nudischer, Lars M. Blank, Vanessa Baier, Stephane Heymans, Florian Caiment, Adrian Roth, David A. Fluri, Jens M. Kelm, José Castell, Nathalie Selevsek, Ralph Schlapbach, Hector Keun, James Hynes, Ugis Sarkans, Hans Gmuender, Ralf Herwig, Steven Niederer, Johannes Schuchhardt, Matthew Segall, Jos Kleinjans

**Affiliations:** 1 Institute of Applied Microbiology, RWTH Aachen, Germany; 2 Roche Pharmaceutical Research and Early Development, Roche Innovation Center Basel, Basel, Switzerland; 3 Department of Cardiology, Cardiovascular Research Institute Maastricht, Maastricht University, Maastricht, Netherlands; 4 Department of Cardiovascular Sciences, Leuven University, Leuven, Belgium; 5 Department of Toxicogenomics, Maastricht University, Maastricht, Netherlands; 6 InSphero AG, Schlieren, Switzerland; 7 Instituto de Investigación Sanitaria. Hospital Universitario La Fe, Valencia, Spain; 8 Functional Genomics Center Zurich, ETH Zurich and University of Zurich, Zurich, Switzerland; 9 Division of Cancer, Department of Surgery and Cancer, Imperial College London, London, UK; 10 Luxcel Biosciences Ltd, Cork, Ireland; 11 European Molecular Biology Laboratory, Cambridge, UK; 12 Genedata AG, Basel, Switzerland; 13 Department Computational Molecular Biology, Max-Planck-Institute for Molecular Genetics, Berlin, Germany; 14 Department of Imaging Sciences and BioMedical Engineering, King’s College London, London, UK; 15 Microdiscovery GmbH, Berlin, Germany; 16 Optibrium Ltd, Cambridge, UK

## General introduction

For more than a decade pharmaceutical R&D has been hampered by considerable attrition rates during clinical trials. The main reasons for drug failure is related to the lack of efficacy, limitations with respect to ADME (absorption, distribution, metabolism and excretion) properties, and—in approximately 30% of the cases—unforeseen toxicity (Kola and Landis 2004). The majority of adverse drug reactions observed in the clinical phase refer to organ injuries, e.g. of the cardiovascular system, the liver, the central nervous system and skeletal muscle (Cook et al. 2014). This clearly demonstrates the limited predictive accuracy of current preclinical models such as the rodent bioassay in evaluating repeated dose toxicity for predicting human toxic risks. It has been argued that overall, only 43% of toxic effects in humans may be correctly predicted by applying rodent-based safety evaluation protocols due to the fact that these assays tend to generate relatively large numbers of false negative as well as false positive read outs (Hartung 2009).

## Toxicological dose descriptors

Obviously, to some extent inter-species differences in toxicant susceptibility may account for this lack of predictive accuracy of preclinical animal models. This consideration has initiated tremendous global efforts in developing alternative approaches for evaluating chemical safety such as Tox21 in the USA, TG-GATEs in Japan, and, within the EU, amongst others, the SEURAT programme, all of which use in vitro human cell models for developing accurate and non-animal based assays for predicting human organ toxicity risks.

A second argument for explaining the observed lack of predictive capacity of animal toxicity models is attributable to the application of fairly high doses of the compound in rodent studies (the OECD Test Guideline for the 28 days repeated dose toxicity study requires a highest dose level which should induce toxic effects but not yet death or severe suffering) which are unlikely to be reached in patients during clinical trials of new drug candidates, or, upon market introduction, in drug-treated patients. Moreover, it has been criticized that in general, in vitro models for assessing toxicity also tend to apply relatively high incubation concentrations of test compounds which do not reflect blood levels achieved in experimental animals, or in patients, for assessing toxicity (Wambaugh et al. 2015). In order to cope with this discrepancy, currently ongoing EU research programmes, e.g. HeCaToS and EUToxRisk, aim to apply physiologically relevant toxicant doses in vitro calculated from—preferably human—kinetic data of the compounds under investigation.

There are already numerous toxicological dose descriptors such as the maximum plasma concentration (C_max_), the average concentration across time (C_average_) or the area (integral) under the concentration–time curve (AUC) (Muller and Milton 2012) which all compare drug exposure to the intensity of specific adverse events. The establishment of such concentration–response correlations requires the systematic application of escalation studies to characterize the dose-dependent effect of a toxicant. However, toxicological dose descriptors inevitably reflect an underlying experimental setup, for example the drug concentration in the incubation media or the duration of drug exposure in an in vitro assay. In this regard, it should also be noted that toxicological dose descriptors usually quantify pharmacokinetic (PK) drug exposure in the venous plasma which is the routine sampling site in clinical practice. However, these plasma concentrations are only surrogate markers for the actual tissue level where the adverse event ultimately occurs. Depending on the physicochemistry or the biological properties of a particular drug such tissue levels may differ significantly in different organs. Alternatively, physiologically based pharmacokinetic (PBPK) models provide a possibility to describe the physiology of the body at a large level of detail. Different organs are explicitly represented in PBPK models to account for their specific physiological role in drug ADME (Kuepfer et al. 2016). The organs are further subdivided into the intracellular and the interstitial space as well as into blood plasma and red blood cells, respectively. Mass transfer inbetween the different sub-compartments is estimated from physicochemical properties of the drug such as the lipophilicity or the molecular weight. The simulation of drug concentration profiles in specific organ compartments allows predicting the concentration profile in the extracellular environment which corresponds to either the interstitial space of an organ or the incubation media of an assay (Hamon et al. 2015). Notably, in vitro–in vivo correlations are directly possible through this equivalence of drug exposure in the assay and the PBPK model, respectively. Likewise, in vitro dose descriptors may be directly translated to an in vivo situation to allow for the application of pharmacokinetics/pharmacodynamics (PK/PD) concepts (Derendorf and Meibohm 1999). The concept of model-based assay design developed in HeCaToS (Hepatic and Cardiac Toxicity Systems modelling) will be introduced in the following.

## Model-based assay design

**Fig. 1:**
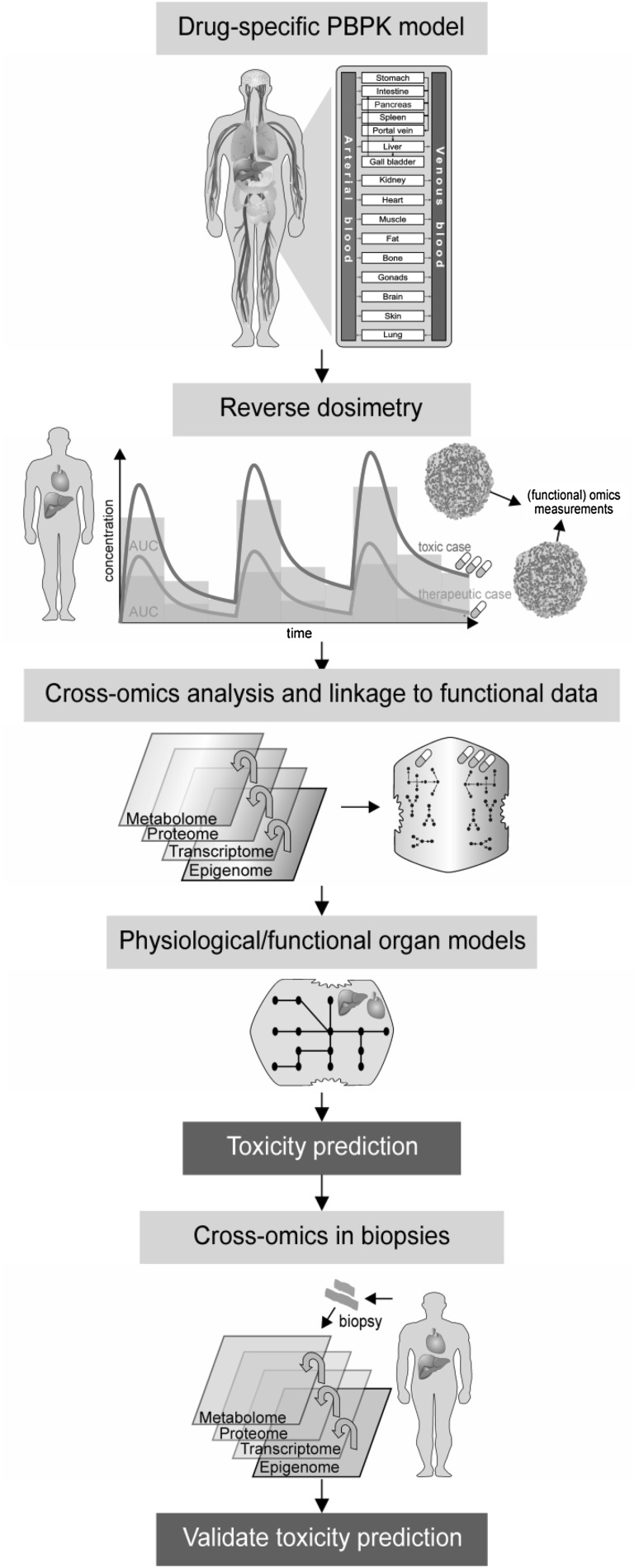
PBPK-based experimental design for cross-omics analyses in 3D liver and heart microtissues

In an initial step, PBPK models are established for sets of different hepato- and cardio-toxicants and are validated based on literature PK data (Fig. 1). Both a therapeutic and a toxic drug dose are then simulated for multiple administrations of up to two weeks of treatment. The therapeutic dose, representing the ‘no adverse effect level’, is selected according to the drug label. The toxic dose in turn is based upon specific in vitro toxicity markers (e.g. IC20). Within HeCaToS, 3D liver and heart microtissues are used to account for different organ specific manifestations of drug-induced injuries such as cardiomyopathy, mitochondrial dysfunction or cholestasis (Beauchamp et al. 2015; Proctor et al. 2017). The drug-specific PBPK models are used to simulate concentration–time curves in the interstitial compartment of the liver or the heart. The profiles are then discretized at multiple sampling times according to a pre-defined, PK-driven experimental schedule. This allows us to approximate the simulated PK profile through physiologically relevant incubation concentrations which in turn requires replacement of the drug-containing assay medium after 2 h, 8 h and 24 h each day. Although this is a labour-intensive approach, the concept allows the unbiased analysis of the emergence of drug-induced side effects as a function of both time and dose without the prior selection of a specific toxicity descriptor. Moreover, the in vitro assay mimics the dynamics of actual PK profiles (including tissue accumulations of drugs following repeated dosing) and thus the tissue exposure in a real patient through the preparatory PBPK simulations. Such highly specific tissue concentrations cannot be analyzed in vivo since this would require invasive sampling. At each time point, 3D microtissues are harvested and cross-omics analyses are subsequently performed to track the emergence of toxic effects at different levels of cellular regulation. This includes epigenomics, transcriptomics, proteomics and metabolomics measurements, as such representing drug-induced physiological endpoints which are further used to characterize induction of toxicity-related pathways and to populate different computational models. In this regard, comparison of ‘omics data from 3D liver and heart microtissues challenged with either the therapeutic or the toxic dose allow the identification of drug-induced pathway responses in a time- as well as a dose-dependent manner. To validate the in vitro findings the ‘omics data generated are matched with ‘omics and clinical data from heart of liver biopsies taken from patients treated with the same drugs and showing symptoms of target organ toxicity as well as clinical data. The comparison of the cross-omics data from the PBPK-based 3D microtissue assay with analogous in vivo data from actual patient biopsies will allow a rigorous assessment whether patterns of acute drug-induced toxicity in patients can actually be reproduced in the lab.

In summary, the HeCaToS project aims to establish better prediction models for human heart and liver toxicity, by challenging 3D human cardiac and hepatic cell models with physiologically relevant doses of cardio- and hepatotoxicants mimicking in vivo PK profiles. The comprehensive analysis of multi-scale deregulation of cell function through cross-omics approaches compared with molecular data from heart or liver biopsies from patients treated with the same toxicants for model validation can be expected to significantly enhance the relevance and predictivity of in vitro preclinical assays in the near future.
